# Enhanced CNN for induction motor fault diagnosis via multi-source data fusion

**DOI:** 10.1371/journal.pone.0330761

**Published:** 2025-08-22

**Authors:** Likang Bo, Meng Li, Hua Tan

**Affiliations:** Hebei Vocational University of Technology and Engineering, Xingtai, Hebei , China; University of Baghdad, IRAQ

## Abstract

This study addresses the challenge of low recognition precision in single fault signal isolation for induction motors by proposing a novel fault diagnosis strategy that integrates multi-source information and an enhanced Convolutional Neural Network (CNN). This approach aims to mitigate the effects of strong nonlinear correlations inherent in fault characteristics. Initially, vibration and stator current signals are preprocessed using denoising autoencoders to improve signal quality. Subsequently, multi-source homogeneous data are fused at the data layer using a correlation variance contribution rate method, effectively integrating information from disparate sources. The fused signals are then transformed into two-dimensional images, serving as input for a refined CNN architecture designed to handle heterogeneous data integration and feature extraction. Finally, the proposed adaptive CNN fault diagnostic model is evaluated using induction motor test datasets. Empirical results demonstrate the method’s ability to effectively utilize both redundant and complementary information from multiple sources and to model the nonlinear dynamics of feature datasets. Specifically, the proposed method achieves an average diagnostic accuracy of 99.0% at 1800 r/min and 94.8% at 2400 r/min, significantly outperforming traditional methods under the same conditions. Furthermore, when compared to other advanced multi-source fusion techniques, the proposed method demonstrates superior performance. These results highlight highlights its potential as a robust tool for induction motor fault diagnosis.

## 1 Introduction

Induction motors are widely used in industrial systems, making accurate and timely fault diagnosis essential for maintaining operational reliability [1]. Traditional diagnostic methods, such as vibration monitoring and current analysis, typically rely on single-sensor input and suffer from limitations like sensitivity to noise, low signal quality, and poor adaptability under variable operational conditions [2–4]. Moreover, effective fault signature extraction is critically hampered by strong background noise in industrial environments. Advanced denoising techniques, particularly stochastic resonance (SR) methods have emerged to amplify weak fault characteristics obscured by noise. For instance: SR-based filter banks [5] leverage noise energy to enhance multi-harmonic fault features in machinery. Fractional nonlinear SR systems [6] boost weak signal detection via adjustable potential-well widths for mechanical faults. Harmonic-Gaussian double-well SR [7] improves fault signature extraction in nonlinear dynamics contexts. However, these methods often depend on manual parameter tuning, limiting their generalizability and adaptability.

To address these challenges, multi-sensor systems and multi-feature fusion strategies have been explored [8,9]. Such approaches allow the integration of complementary information from heterogeneous sources, resulting in more reliable diagnostic results. At the same time, machine learning techniques, particularly deep learning models, have shown promise in processing high-dimensional data for fault classification. Unlike shallow models that rely on manual feature extraction [10], deep learning networks like CNNs offer powerful automatic feature extraction capabilities [11,12]. Nevertheless, most existing multi-source fusion techniques—such as direct signal stacking—fail to fully leverage the interrelationships among data streams, leading to information loss and reduced diagnostic accuracy [13].

This paper proposes a novel fault diagnosis framework that combines multi-source information fusion with an adaptive convolutional neural network (CNN) to improve classification performance for induction motor faults. First, vibration and current signals are denoised using a denoising autoencoder. Then, homogeneous signals are fused using the correlation variance contribution rate method, while heterogeneous features are integrated through an adaptive CNN structure capable of dynamically adjusting its depth based on convergence performance. This method enhances the representation and utilization of both redundant and complementary signal characteristics, leading to more accurate fault identification.

The main innovations and contributions of this study are as follows:

1) A novel multi-source information fusion strategy is proposed, combining data-layer fusion of homogeneous signals using the correlation variance contribution rate method and feature-layer fusion of heterogeneous signals, enabling comprehensive utilization of sensor data.
2) An adaptive Convolutional Neural Network (CNN) architecture is introduced, which dynamically expands the network structure based on convergence speed, thereby improving fault feature learning and classification performance while avoiding overfitting.
3) A signal-to-image conversion technique is employed to transform 1D time-domain sensor data into 2D grayscale images, enhancing the ability of the CNN to extract spatial features directly from raw sensor data.

## 2. State of the art

### 2.1 Induction motor fault diagnosis method

Fault diagnosis refers to a spectrum of methodologies centered around the detection, identification, and prognostication of anomalous states within a system. This diagnostic procedure can be systematically delineated into three foundational stages as delineated in the literature [14]. Firstly, there is the acquisition of irregular signals emanating from equipment during malfunction periods, encompassing a variety of indicators such as alterations in current and voltage, mechanical oscillations, auditory disturbances, thermal fluctuations, and torque power deviations.

The subsequent stage involves the application of signal processing techniques among others to distill salient features from the aforementioned abnormal signals, representing the fault characteristics effectively. This phase leverages both one-dimensional and two-dimensional data representations to encapsulate the critical attributes reflective of the fault dynamics.

In the final stage, a judicious analysis is conducted utilizing the extracted features in conjunction with other pertinent information to adjudicate the current state of the equipment. This facilitates the stratification and localization of the fault, culminating in the execution of a diagnostic operation aimed at identifying the fault with precision. This comprehensive process thus strives to ensure a meticulous fault diagnosis operation, engendering an enhanced understanding and management of equipment conditions.

**Fig 1:**
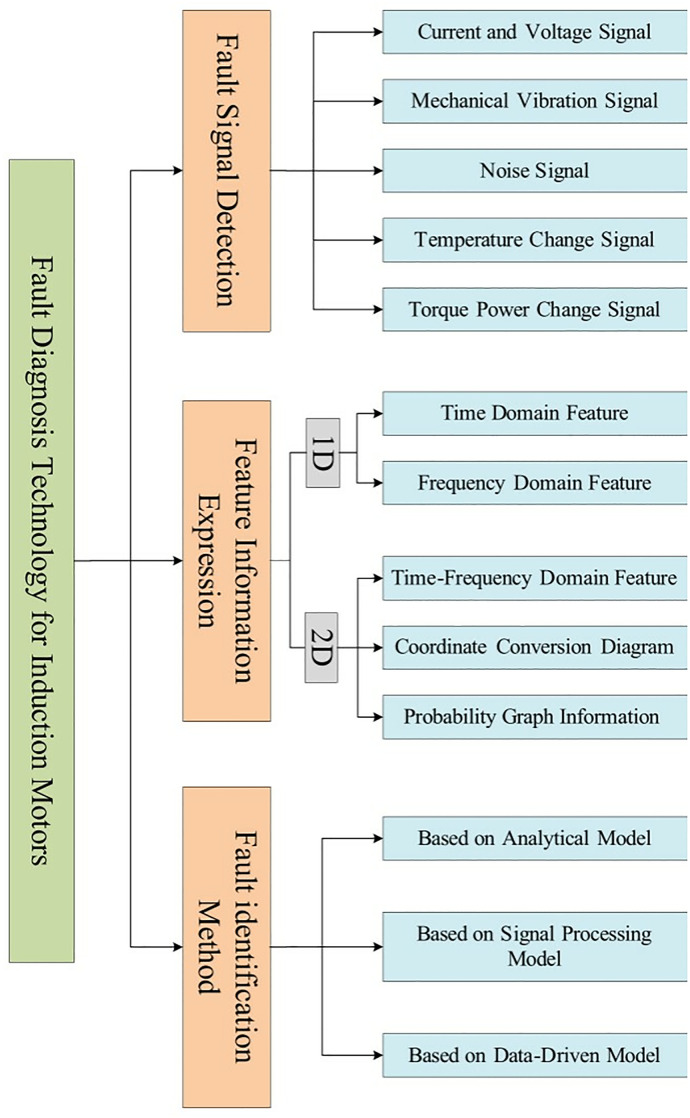
Motor fault diagnosis method.

From an analytical standpoint, fault diagnosis methodologies can be broadly segmented into three principal categories as illustrated in Fig 1: analytical model-based techniques, signal processing-based strategies, and data-driven approaches.

The first category, analytical model-based strategies, necessitates the formulation of a mathematical representation of the fault within the target of the assessment. This approach is predominantly applicable to motors where ample sensor data is available, facilitating the development of a precise quantitative data model grounded on the mechanistic constitution of the system. Characteristic techniques within this category encompass parameter estimation methods, state estimation methods, and analytical redundancy methods [15]. While these methods hold the potential to generate exact models under optimal conditions, their real-world application poses considerable challenges owing to their complexity and stringent data prerequisites.

Signal processing-based strategies, conversely, rely on the real-time analysis of field-collected signals, which are then juxtaposed against baseline, normal signals to discern potential faults within the motor system [16]. This diagnostic technique provides a method to scrutinize and interpret signal data, highlighting discrepancies that may signal malfunction.

Lastly, the data-driven diagnosis approach leverages contemporary artificial intelligence algorithms — including but not limited to neural networks, fuzzy logic, and deep learning — to inaugurate a diagnostic model through initial training phases followed by the integration of processed fault signals for fault categorization [17]. A notable divergence from conventional motor fault diagnostic processes, which typically initiate with signal preprocessing followed by fault feature extraction, is observed in this approach. Instead, it streamlines the initial steps by implicitly conducting feature selection through the direct input of refined raw signals into a pre-trained model, thus enhancing efficiency and expediting the diagnostic procedure.

### 2.2 Fault diagnosis based on deep learning

In light of advancements in artificial intelligence technology, intelligent fault diagnosis techniques exhibit pronounced superiority compared to conventional fault detection methods, principally due to their foundational reliance on deep learning algorithms. These algorithms, epitomizing the zenith of knowledge-based approaches, obviate the necessity for support grounded in expert experience, engendering fault diagnosis and delineation through nuanced signal feature extraction processes.

Pioneering work in this field is characterized by innovative signal preprocessing and deep learning network combinations. Xiong et al. [18] leveraged the mutual dimensionless theory aligned with analogous Gram matrices to enhance the preprocessing of vibration signals, interfacing with Convolutional Neural Networks (CNN) to facilitate intricate bearing fault classification studies. Parallel efforts by Kumar et al. [19] discerned bearing anomalies from rotor broken bar faults through the deployment of a dilated CNN model. Moreover, the scholarly initiatives of Ribeiro et al. [20] revolved around utilizing vibration time domain signals, harnessing the potential of multi-channel 1D-CNN for the comprehensive multi-fault categorization of motors.

Further complementing this research trajectory, Huang et al. [21] synthesized complementary ensemble empirical modal decomposition and CNNs to accurately diagnose broken bar and bearing faults within asynchronous motors operating under variable frequency environments. A salient challenge in this domain remains the prevalent usage of two-dimensional images as input in traditional CNN architectures, while most fault signals manifest as one-dimensional entities. Addressing this, Yang [22] independently adopted transformation techniques, such as the Short Time Fourier Transform (STFT) and Continuous Wavelet Transform (CWT), to transmute gathered 1D signals into 2D visualizations conducive to CNN integrations.

Recent advancements in deep learning have introduced transformer-based architectures and attention mechanisms into the field of fault diagnosis. For instance, Cen et al. [23] proposed a mask self-supervised learning-based transformer for bearing fault diagnosis with limited labeled samples, achieving high fault classification accuracy for wind turbine blades. Additionally, Sui et al. [24] designed a bearing fault diagnosis method that incorporates attention-based fusion of multi-domain and multi-source information, highlighting the capacity of attention mechanisms to focus on discriminative features. These attention-driven approaches provide enhanced feature learning by dynamically weighting the importance of different input components, thereby improving fault detection performance in complex scenarios. While our proposed model does not explicitly employ transformer or attention modules, it achieves similar adaptive capability through its dynamic CNN expansion strategy, which adjusts the network structure based on convergence behavior. This allows our model to selectively enhance representational depth and learn critical fault features without overfitting.

### 2.3 Fault diagnosis based on multi-source data fusion

Leveraging superior fault tolerance, heightened accuracy, expedited processing capabilities, and pronounced complementarity, multi-sensor data fusion technology is progressively finding applicability and undergoing development in a plethora of sectors. In the context of motor fault diagnosis, this emergent technology is conceptualized as a systematic approach to meticulously analyze fault feature information acquired through a multitude of sensors, aiming to pinpoint the specific type of motor malfunction with a high degree of accuracy.

Serving as a pillar in fault diagnosis, information fusion theory orchestrates the harmonization of diverse information sources, data stemming from a variety of classifiers, and the meticulous utilization of several dimensions spanning both feature and decision layers. A host of researchers have made significant strides in this domain, evolving sophisticated methodologies to enhance diagnostic precision. Zhang et al. [25] innovated by deriving a fusion correlation spectrum from current and vibration signals, a strategy that amplified fault characteristic frequencies while mitigating irrelevant components present in the frequency spectrum. Parallelly, Li et al. [26] employed a Bayesian network fortified with enhanced Dempster-Shafer theory to execute hub motor fault diagnosis leveraging vibration and noise signals. In a similar vein, Zhao et al. [27] orchestrated fault diagnosis by overlaying filtered sound and vibration signals, utilizing 1D-CNN as a diagnostic tool. Yuan et al. [28] enhanced diagnostic velocity and accuracy for motor bearing faults through isolated extraction and 1D-CNN fusion of features from vibration and instantaneous speed signals. Meanwhile, Zhou et al. [29] advanced the field by transmuting multi-sensor vibration signals into images, followed by feature fusion through CNN architectures.

**Fig 2:**
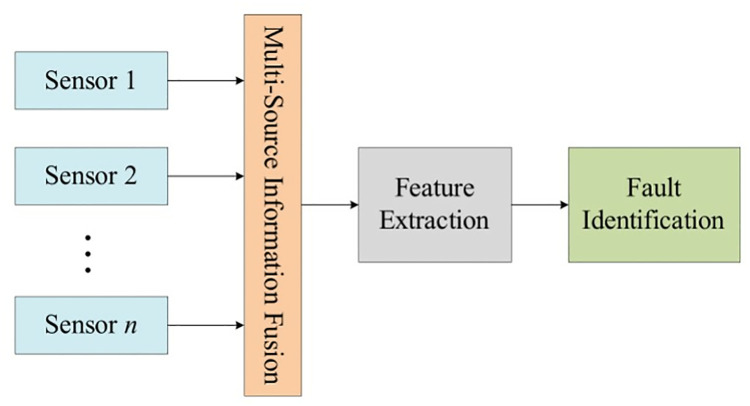
Data layer fusion structure diagram.

Broadly, data fusion methodologies can be delineated into three distinct yet interconnected forms delineated by the fusion object or the procedural stage at which fusion is undertaken, namely: data layer fusion, feature layer fusion, and decision layer fusion, as elucidated in [30]. Fig 2 elucidates the structure of data layer fusion, which refrains from altering signal data harvested by sensors, instead opting for a direct fusion of sensor outputs. After this process, pertinent features are derived from the fusion results to facilitate fault identification. Despite offering an avenue that prevents extensive data alteration and thereby minimizing information loss, this structure demands a high sensor homogeneity and tolerates a diminutive rate of information dissipation. However, it grapples with challenges including limited fault tolerance and heightened computational demands, necessitating further refinements for optimal functionality.

**Fig 3:**
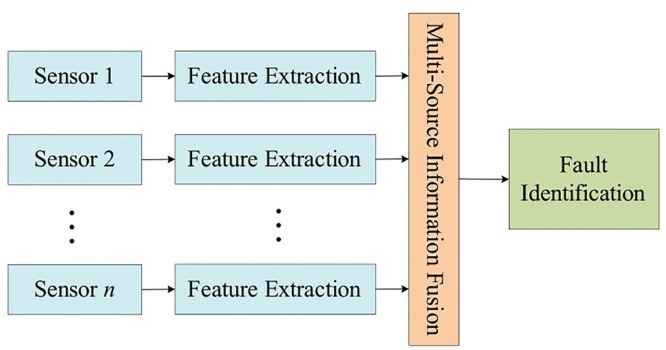
Feature layer fusion structure diagram.

Fig 3 shows the feature layer fusion structure. This structure first needs to extract the feature information of the signals collected by each sensor, and fuse this feature information, and identify the fault information according to the fusion result. This structure does not require homogeneity of each sensor, and the system is more fault tolerant and less computationally intensive, but there is information loss during feature extraction.

**Fig 4:**
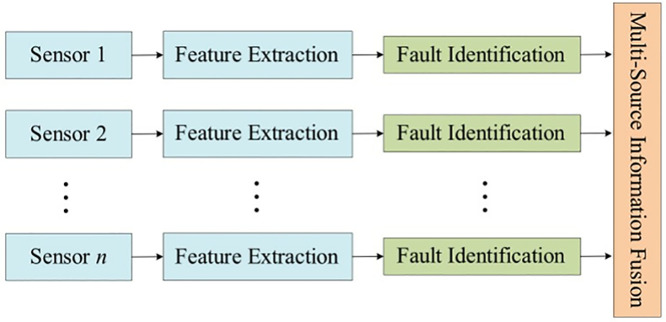
Structure of decision level integration.

Fig 4 shows the structure of decision layer fusion, which processes all sensor information individually, extracts feature information and identifies them separately, and fuses the results of the individual identifications to perform fault discrimination. This structure has the largest amount of loss of original information and is more demanding for pre-feature extraction and fault identification, but is more fault-tolerant and has the least amount of computation.

Feature fusion has stronger generality than data layer fusion and can be used for multi-source similar or heterogeneous information fusion. Moreover, compared with decision layer fusion, feature fusion layer retains more original information, and its results are more credible than decision fusion results. Therefore, in this paper, based on the deep learning-based motor fault diagnosis model, we combine data layer and feature layer fusion theory to reduce the difficulty of deep network training on the one hand, and improve the diagnostic efficiency while improving the diagnostic accuracy on the other hand.

## 3 Motor fault diagnosis based on multi-source information fusion

### 3.1 Motor multi-source information composition

The main types of faults in induction motors include electrical and mechanical faults. To achieve accurate identification of motor faults, it is firstly necessary to analyze the fault mechanism of the motor.

#### 3.1.1 Motor fault mechanism based on stator current.

When a short-circuit fault occurs in the stator winding, the symmetry of the stator winding is damaged and the odd harmonics of its current components, especially the 5th harmonic, are enhanced by the asymmetry of the three-phase winding. The characteristic frequency of stator current is expressed as


$$f_{\text{s}}=|[n\pm 2k(1-s)]f\;|$$ (1)

Where, *f* is the power supply frequency. *s* is the induction motor turndown rate. *n*, k = 1,2,3,....

When there is a stator short circuit fault in the motor, analysis of the stator current spectrogram will reveal that the amplitude at the characteristic frequency in the stator current spectrogram during the stator short circuit fault state is larger than that in the normal state, and the deviation is proportional to the severity of the fault, so that the stator short circuit fault can be identified. When there is a broken rotor bar fault, there will be a sideband in the stator current spectrum at 2s from the power frequency. The principle for identifying rotor broken bar faults based on stator current is that there are characteristic frequencies in the current spectrum as shown in the following formula.


$$f_{tz}=(1\pm 2s)f$$ (2)

#### 3.1.2 Motor failure mechanism based on vibration signal.

When there is no fault in an induction motor, the stator seat is subjected to rotational forces and vibrates at a frequency of *2f.* However, when there is a stator winding fault in the induction motor, the vibration signal of the base will be enhanced. Besides the normal frequency, there will also be harmonic components such as 4*f*, 6*f*, 8*f*, etc. The vibration accelerometer detects the characteristic frequency of the vibration signal of the motor base to determine whether the motor has a stator winding fault.

When the induction motor has a rotor eccentricity fault, there are two characteristic frequencies in the vibration signal of the machine base, namely the static eccentricity characteristic frequency *f*_*jt*_ and the dynamic eccentricity characteristic frequency *f*_*dt*_. The values of the two characteristic frequencies are


$$f_{jt}=2f$$ (3)

$$f_{dt}=2sf$$ (4)

When there is a fault in the motor bearing, the frequency spectrum of the housing vibration signal will contain the corresponding characteristic frequency components, and the characteristic frequencies presented by different fault types are different. Among them, the bearing outer ring fault frequency *f*_*od*_, inner ring fault frequency *f*_*id*_, ball fault frequency *f*_*bd*_, cage fault frequency *f*_*cd*_ are defined as


$$f_{\text{od}}=\frac{l}{2}f_{\text{rm}}\left(1-\frac{d_{\text{b}}}{d_{\text{p}}}\text{cos}\;\beta \right)$$ (5)

$$f_{\text{id}}=\frac{l}{2}f_{\text{rm}}\left(1+\frac{d_{\text{b}}}{d_{\text{p}}}\text{cos}\;\beta \right)$$ (6)

$$f_{\text{bd}}=\frac{d_{\text{p}}}{2d_{\text{b}}}f_{\text{rm}}[\left(1-{\left(\frac{d_{\text{b}}}{d_{\text{p}}}\text{cos}\;\beta \right)}^{2}\right]\;$$ (7)

$$f_{\text{cd}}=\frac{1}{2}f_{\text{mm}}\left(1-\frac{a_{\text{b}}}{d_{\text{p}}}\text{cos}\;\beta \right)$$ (8)

Where, *f*_*rm*_ is the motor rotation frequency. *d*_*b*_, *d*_*p*_ are the bearing rolling body diameter and cage diameter respectively. *l* is the number of bearing rolling bodies. *β* is the rolling element contact angle.

#### 3.1.3 Multi-source information composition.

In the data acquisition layer, 2 types of sensor information are obtained using vibration sensors and current sensors. The multi-source sensor information of induction motors can be divided into two categories: homogeneous information and heterogeneous information. Multi-source homogeneous information refers to the monitoring of multiple similar sensor information of the same component, such as motor vibration including horizontal vibration signal *u*_*h*_ and vertical vibration signal *u*_*v*_. Similar sensor signals are fused into one signal by the data layer, and the fused signal constitutes one type of multi-source heterogeneous information. Multi-source heterogeneous information refers to the monitoring information of different sensors, including fused vibration signal *u*_*f*_ and stator current signal *i*_*m*_.

### 3.2 Deep learning-based induction motor fault diagnosis

#### 3.2.1 Data denoising.

**Fig 5:**
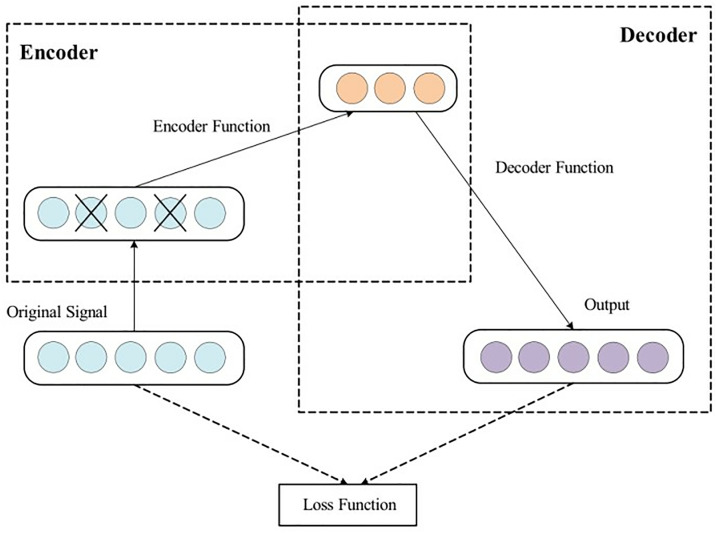
Denoising autoencoder network framework.

The data collected directly from induction motor sensors are extremely nonlinear, and the noise pollution of sample data caused by the operating environment makes it difficult to dig out the deep relationships in the data information during fault diagnosis. Therefore, to improve the diagnostic accuracy, the denoising autoencoder is used to reduce the stator current and vibration signals of induction motors. First, the autoencoder performs a certain degree of noise separation on the original data. Then, the data are fed into the encoder and decoder modules of the denoising autoencoder network. The encoder module eliminates the effect of noise based on the statistical properties of the data. The decoder module then reconstructs the data according to the coding laws. Fig 5 shows the framework of denoising autoencoder.

#### 3.2.2 Multi-source information fusion framework.

**Fig 6:**
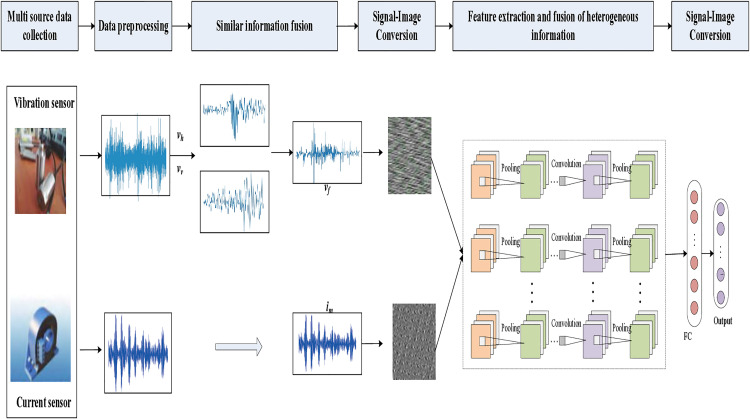
Multi-source information fusion framework.

The traditional multi-source information fusion mode is to serially superimpose multiple signals into one signal, and this fusion mode does not fully utilize the correlation and complementarity among the signals. Since there are many induction motor monitoring signals with both homogenous and heterogeneous information from multiple sources, the comprehensive and effective use of this information is important to improve the diagnostic accuracy of induction motors. Therefore, a multi-source information fusion model is proposed. The correlation variance contribution rate method is used to achieve the fusion of homogenous information of induction motors in the data layer. Multiple signals of the same type are fused into one signal for heterogeneous fusion. Then, the features of each heterogeneous information are extracted and feature fusion is completed using adaptive CNN network. The fusion framework is shown in Fig 6.

The adaptive CNN structure in our framework is designed to dynamically expand its branches based on the convergence speed of the network, allowing it to adapt to the complexity of the fault diagnosis task. This adaptability is controlled by two key parameters: $r_{max}\;$ the maximum number of branches, and $c_{h}$, the convergence threshold. We conducted preliminary experiments to evaluate different values for these parameters and found that setting $r_{max}$ higher than 5 resulted in diminishing returns in diagnostic accuracy while increasing training time and computational complexity. Conversely, setting $r_{max}<\text{3}$ led to underfitting and insufficient feature learning. Similarly, the convergence threshold Ch=0.1 was selected as a balance point: a stricter threshold (e.g., 0.05) delayed convergence unnecessarily, while a looser threshold (e.g., 0.2) prematurely halted learning before optimal performance was reached. Therefore, the current parameter choices were determined through iterative experimentation to ensure model efficiency and classification robustness.

(1) **Same type of information data layer fusion**

Data layer fusion directly fuses raw data of homogeneous information and can provide much subtle information. The correlation variance contribution ratio method effectively exploits the correlation and complementarity between the signals of sensors. This method uses variance contribution rate as the basis to allocate fusion coefficients based on the importance of the correlation between information. Multiple signals of the same type are fused into one signal, achieving dynamic fusion between similar information, thereby avoiding leakage and loss of effective information.

Let *x*(*n*) and *y*(*n*) be 2 deterministic signals with finite energy. Then the correlation coefficients of signals *x*(*n*) and *y*(*n*) are


$$\rho_{xy}=\frac{\sum_{n=0}^{\infty }\;x(n)y(n)}{{\left[\sum_{n=0}^{\infty }\;x^{2}(n)\sum_{n=0}^{\infty }\;y^{2}(n)\right]}^{\frac{1}{2}}}=\frac{R_{xy}}{\overset{¯}{E_{x}E_{y}}}$$ (9)

Where, $\overset{¯}{E_{x}E_{y}}$ is the square root of the product of the respective energies of the signals *x*(*n*) and *y*(*n*), and its value is constant. Therefore, the correlation coefficients $\rho_{xy}$ of *x*(*n*) and *y*(*n*) is determined by the correlation coefficient *R*_*xy*_.

Suppose *m* similar sensor signals *x*_1_ *(n*), *x*_2_ *(n*),..., *x*_*m*_ *(n*), then the mutual correlation of signals can be expressed as


$$\begin{array}{c} R_{x_{i}x_{j}}(t)=\frac{1}{n-t}\sum_{t_{0}=1}^{n-t}\;x_{i}\left(t_{0}\right)x_{j}\left(t_{0}+t\right) \end{array}$$ (10)

Where, *n* is the number of data points. *t* denotes the signal time series. The total correlation energy of the *ith* sensor with all similar sensor signals can be expressed as.


$$\begin{array}{c} E_{i}=\sum_{j=1,j\neq i}^{m}\;E_{ij}=\sum_{j=1,j\neq i}^{m}\;\sum_{t=0}^{n-1}\;{\left[R_{ij}(t)\right]}^{2} \end{array}$$ (11)

Where, *E*_*ij*_ represents the energy of the signal calculated by correlation. Then, energy normalization is performed on the signals of m similar sensors, and the energy normalization formula is


$$y_{i}=\frac{x_{i}}{\sqrt{E_{i}^{2}}}$$ (12)

Where, *x*_*i*_ is the sequence of data signals collected by the *ith* sensor at a certain sampling frequency in time *T*. The variance contribution rate of *n*-dimensional energy normalized signal *y*_*i*_ is


$$K_{il}=\frac{{\left[y_{i}(l)-\mu_{i}\right]}^{2}}{n\sigma_{i}^{2}}$$ (13)

Where, *y*_*i*_ *(l*) represents the *lth* point of $\mu_{i}$ and $\sigma_{i}^{2}$ are the mean and variance of the energy normalized signal *y*_*i*_.

From the variance contribution of similar signals collected by *m* similar sensors at a given moment, the distribution coefficient of *x*_*i*_ (*l*) is obtained as


$$k_{il}=\frac{K_{il}}{\sum_{i=1}^{m}\;K_{il}}$$ (14)

Based on the obtained allocation coefficient, *m* sensor signals are fused in the same category. The value of the *lth* data point of the resulting signal *x* after fusion is


$$\begin{array}{c} x(l)=\sum_{i=1}^{m}\;k_{il}y_{i}(l) \end{array}$$ (15)

(2) **Signal-image conversion**

The traditional data-driven fault diagnosis method requires high original signal feature extraction, relies on the experience and technology of technicians, and is complex and time-consuming. In addition, there are problems such as missing signal features, poor generalization ability and local optimal solutions, which affect the final classification results. CNN can directly learn the original signal and automatically complete feature extraction as well as fault identification, and has the advantages of stronger ability to extract two-dimensional data features and fewer network parameters, which are widely used in the field of image recognition.

The original stator current and vibration signals of induction motors are one-dimensional time-series signals, and the difference between two-dimensional images and one-dimensional time-series signals causes it to be ineffective in industrial applications. Therefore, based on the characteristics of industrial signals, applying two-dimensional CNN to the field of motor fault diagnosis is a new breakthrough point. This article adopts the signal image conversion method to convert one-dimensional temporal signals into two-dimensional grayscale images. Its advantage is to eliminate expert experience as much as possible. And it maximizes the preservation of the features of the original signal, fully leveraging the advantages of two-dimensional convolutional neural networks in image recognition.

**Fig 7:**
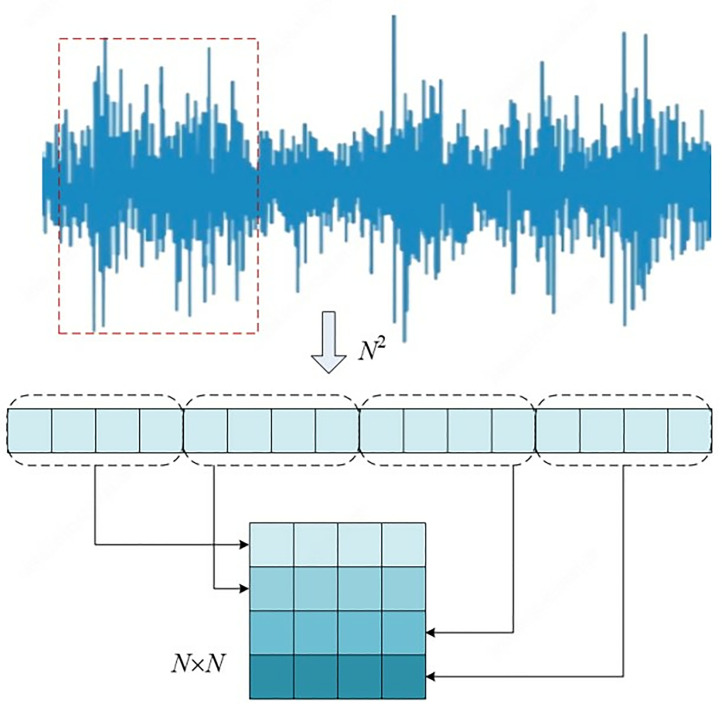
Signal-image conversion principle.


The signal-to-image conversion method is shown in Fig 7. A two-dimensional grayscale image of **N* *× *N* is obtained by randomly selecting signals of length *N*^2^ from the original one-dimensional time-domain signal and filling the pixels of the image row by row each time the signal of length *N is selected.* Since the pixel values of grayscale images are 0–255, it is also necessary to normalize the data of each pixel point using formula (16).

$$L(i,j)=\text{round}\left\{\frac{Q((i-1)\times N+k)-min(Q)}{max(Q)-min(Q)}\times 255\right\}$$ (16)

Where, *Q*(*i*), i = 1,2,..., *N*^2^ denotes the value taken for each point in the intercepted 1D signal. *l*(*i*, *j*) denotes the pixel value of each point in the 2D grayscale image.

In our approach, the fused 1D signals are converted into 2D grayscale images, which serve as input to the adaptive CNN. This choice of representation was made for several key reasons, which distinguish it from more common methods such as spectrograms, Continuous Wavelet Transform (CWT), or recurrence plots.

First, converting 1D signals directly into 2D grayscale images preserves the complete temporal sequence of the data without the need for complex transformations. This is crucial for time-series analysis in fault diagnosis, as it ensures that the temporal dependencies and patterns in the signal are maintained. In contrast, methods like spectrograms require selecting a window size, which can distort the time-frequency resolution and potentially obscure critical fault signatures. Similarly, CWT, while powerful for multi-resolution analysis, introduces computational complexity and redundancy in the feature space. Recurrence plots, although useful for visualizing dynamical systems, do not directly translate into a format that leverages the spatial feature extraction strengths of CNNs. Second, grayscale images are computationally efficient to generate and process, making them well-suited for real-time or resource-constrained diagnostic systems. Unlike CWT or spectrograms, which involve computationally intensive transformations, our method simply reshapes the 1D signal into a 2D grid and normalizes the values to the grayscale range (0–255). This simplicity reduces preprocessing time and allows the CNN to focus on learning spatial hierarchies directly from the raw data. Third, CNNs are inherently designed to excel at extracting spatial features from image data. By representing the signals as 2D images, we enable the CNN to apply its convolutional filters across both the time and amplitude dimensions, capturing patterns that might be indicative of specific faults. This direct mapping from signal to image avoids the information loss or alteration that can occur with frequency-domain transformations, ensuring that all original data points are preserved for the model to learn from. Finally, grayscale images offer an additional benefit in terms of interpretability. Unlike abstract representations such as spectrograms or recurrence plots, grayscale images can be visually inspected, providing an intuitive way to understand the model’s behavior.

(3) **Fusion of heterogeneous information feature layers**

**Fig 8:**
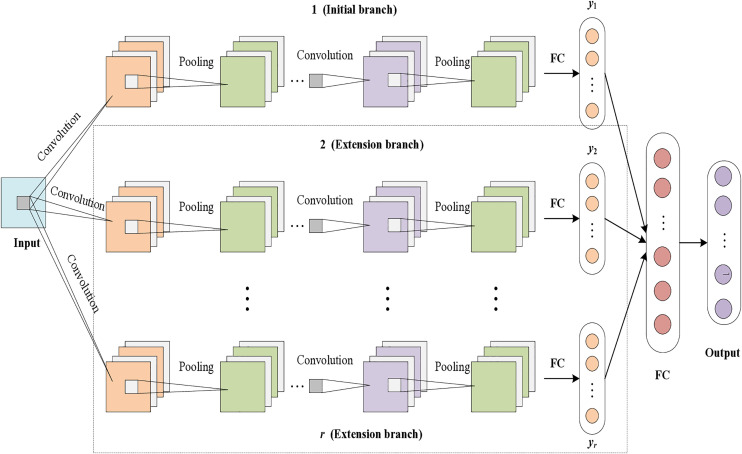
Adaptive CNN network structure.

Adaptive CNN can optimize the network hierarchy according to the actual network performance, take the convergence speed as the evaluation index, and adaptively expand the network to extract the representative fault features of each multi-source information. In this paper, we use adaptive CNN for feature extraction and fusion of heterogeneous information, and its network structure is shown in Fig 8. The adaptive CNN structure proposed in this article draws inspiration from the HACNN architecture in reference [31]. However, the difference is that the literature [31] uses the one-dimensional CNN model to adaptive learning the vibration signal and current signal under each health conditions, and its output values is the difference feature after heterogeneous fusion rather than the diagnosis result. The proposed method adopts a 2D CNN model in this paper, which inputs two-dimensional images of vibration signals and current signals under different health states, and outputs the final diagnostic results using the Softmax function.

The convolutional layer is the core part of the whole network, consisting of multiple feature maps, each of which is connected to a local region of the feature map of the previous layer through a convolutional kernel, and the data features are extracted by convolutional filtering of each input feature map. The mathematical model of the convolutional layer can be expressed as


$$\begin{array}{c} X_{j}^{l}=f\left(\sum_{i\in M_{j}}\;X_{i}^{l-1}*K_{ij}^{l}+b_{j}^{l}\right) \end{array}$$ (17)

Where, *M*_*j*_ is the input feature map. $X_{j}^{l}$ is the *jth* output feature map of the *l*th layer. $K_{ij}^{l}$ is the convolution kernel. $b_{j}^{l}$ is the bias. *f*(.) is the activation function.

The pooling layer reduces the data dimensionality by scaling the mapping of the feature map of the previous layer, and improves the robustness of the network. Among them, the maximum pooling function is the most used pooling function, and its mathematical expression is


$$P_{j}=\underset{j\in S}{max}\;X_{j}$$ (18)

Where, *S* is the pooling window size. *P*_*j*_ denotes the *jth* output of the pooling layer.

The adaptive CNN network has a total of *r* branches, containing initial branches and extended branches. The initialized network contains only one initial branch. Adaptive extension refers to extending a new branch with the same structure and parameters as the initial branch on the basis of the initial network. When the convergence speed meets the requirements or *r* reaches the maximum value *r*_*max*_ of the preset number of branches, adaptive extension will stop. *r*_*max*_ is set to 5, and the convergence speed *v*_*cs*_ is defined and the requirements should be satisfied as follows:


$$v_{\text{cs}}=e_{pre}-e_{cur}⩾C_{h}$$ (19)

Where, *e*_*pre*_ and *e*_*cur*_ are the average error of the previous network and the current network. *c*_*h*_ is the expected threshold, set to 0.1. The average error of network training is calculated as


$$e_{\text{mean}}=\frac{\sum_{j=1}^{N}\;\sum_{i=1}^{c}\;{\left(y_{i}^{j}-y_{\text{lab}}^{j}\right)}^{2}}{N}$$ (20)

Where, *N* is the number of samples. *c* is the number of categories. $y_{\text{lab}}^{j}$ is the true category label of the training sample.

When performing adaptive expansion, a new fully connected layer is needed to fuse the output results of the initial branch and the extended branch. The output result of output layer activation function fusion is


$$\begin{array}{c} y=f\left(y_{1}+\sum_{k=2}^{r}\;\omega_{k}y_{k}\right) \end{array}$$ (21)

Where, *y*_1_ and *y*_*k*_ are the output results of the initial branch and the extended branch *k,* respectively.

**Fig 9:**
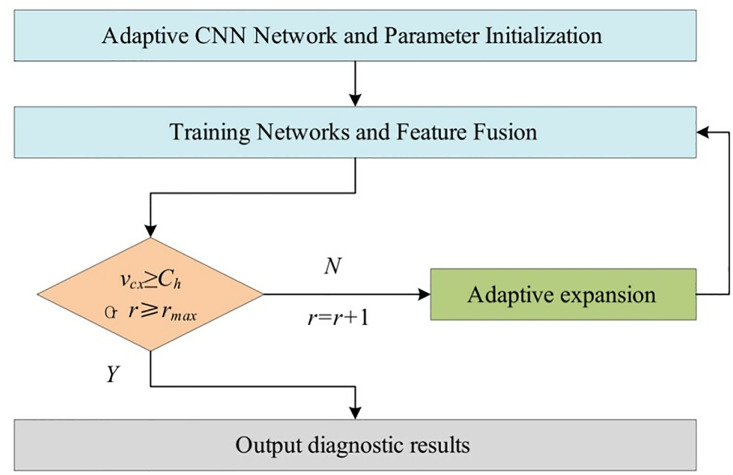
Adaptive CNN network training process.

During the training of the adaptive CNN network, the network structure and related parameters of the initial branch are kept unchanged, and the output result *y*_1_ is kept first, and then fused with the output result *y*_1_ after the training of the extended branch, and the corresponding weights of the extended branch are updated by the BP algorithm, and the adaptive extended learning is completed when all the extended branches are extended. When all the expansion branches are completed, the diagnosis results are obtained through the network output layer. The flow chart is shown in Fig 9.

By adjusting the relevant parameters through repeated experiments, the final structure of the initial branch network model obtained contains four convolutional layers, four pooling layers and two fully connected layers. The parameters of the convolutional layers are shown in Table 1. All convolutional layers have undergone all zero padding processing. Dropout processing is performed after each layer of convolution operation to improve the generalization performance of the network. The pooling layer adopts maximum pooling, with a pooling size of 2. The number of nodes in two fully connected layers is 512 and 4, respectively. The first convolutional layer uses a larger convolutional kernel (16 × 16) can increase the perceptual field of the model input. The smaller convolutional kernels (3 × 3) and deeper network structure are used in layers 2–4 to fully exploit the implied fault features in the signal. The experimental results show that the optimal number of branches *r* for the adaptive CNN network is 3. Each branch has the same structure and parameters as the initial branch.

**Table 1:** Adaptive CNN network parameters.

No.	Network layer	Convolutional kernel size	Number of convolutional kernel
Conv1	Convolutional Layer 1	16 × 16	16
Pool 1	Maximum pooling layer 1	2 × 2	
Conv2	Convolutional Layer 2	3 × 3	16
Pool 2	Maximum pooling layer 2	2 × 2	
Conv3	Convolutional Layer 3	3 × 3	64
Pool 3	Maximum pooling layer 3	2 × 2	
Conv4	Convolutional Layer 4	3 × 3	128
Pool 4	Maximum pooling layer 4	2 × 2	
FC 1	Fully connected layer	512 Nodes	
		ReLU activation function	
FC 2	Softmax	4 Nodes	
		Softmax activation function	

### 3.4 Induction motor fault diagnosis process

The process of induction motor fault diagnosis based on adaptive CNN network is described as follows.

(1) Collecting multiple sensors monitoring information from induction motors using vibration sensors and current sensors, and

Divided into multi-source homogeneous information and multi-source heterogeneous information.

(2) Noise reduction of the original data using a denoising autoencoder.
(3) Data layer fusion is achieved by fusing multiple homogeneous sensor signals from multiple sources of the same kind of information into one signal using the correlation variance contribution rate method.
(4) The signal-image conversion method is used to convert the fused 1D vibration signal and the stator current signal into 2D images respectively.
(5) The training set and test set are set proportionally to the multi-source heterogeneous information fused by the data layer, and the training set is fed into the initial adaptive CNN network with parameter initialization.
(6) Train the network and judge whether the network needs to be adaptively extended according to the convergence speed and the number of branches. If the condition *v*_*cx*_* ≥ C*_*h*_ or *r ≥ r*_*max*_ is satisfied, save the network model parameters and output the diagnosis results. Otherwise, the network continues to be adaptively expanded.
(7) The test set is input to the constructed adaptive CNN and the method is used to implement fault diagnosis of induction motors to verify the effectiveness of the proposed fault diagnosis method.

## 4 Experimental analyses

The environment configuration of the experiments is shown in Table 2.

**Table 2:** Experimental environment configuration table.

Configuration name	Configuration parameter
Operating system	Windows 10 Pro_64
CPU	Intel Core i7-7800X @3.5GHz
GPU	NVIDIA GeForce GTX 1080Ti
Python	3.7
Tensorflow version	2.7.0

To quantify the diagnostic performance of the proposed method on induction motors, the accuracy is used to evaluate the performance of the proposed. To quantify the performance of the proposed method on induction motors, the performance of the proposed fault diagnosis method is evaluated using accuracy.

### 4.1 Signal acquisition

**Fig 10:**
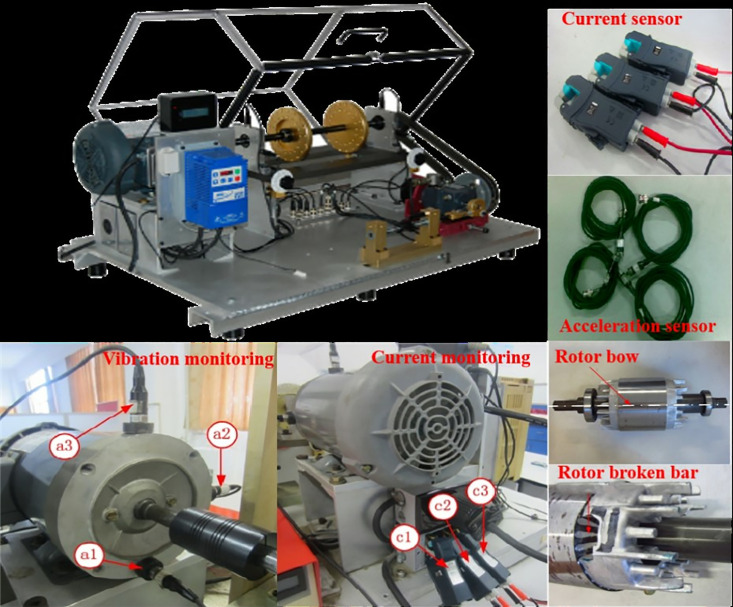
Structure of the experimental platform.

To authenticate the efficacy of the posited methodology for discerning motor compound faults, a sophisticated experimentation regimen was conceived leveraging a meticulously designed test bench that replicates the motor and rotor system intricacies to simulate broken rotor bar faults and accompanying compound faults. This experimental infrastructure, depicted in Fig 10, incorporated an assembly of essential components including a motor, a rotary shaft, an analog load system, acceleration sensors, current clamps, and a data acquisition apparatus.

Ensuring an optimal sensor arrangement in alignment with the dictates of the national standard GB 10068−2000 and grounded in the mechanistic understanding of asynchronous motor failures, the setup included a strategic placement of acceleration sensors — a1, a2, and a3 — in the axial, radial, and vertical orientations at the drive end of the asynchronous motor. This endeavor aimed to bolster the spatiotemporal breadth of the data acquisition system, thereby augmenting the sensitivity and effectiveness in fault signal identification. Furthermore, this intricate arrangement guaranteed the trajectory extension lines from sensors a2 and a3 intersected the focal point of the bearing support, while current sensors labeled c1, c2, and c3 were mounted on the A, B, and C phases of the motor respectively.

Commensurate with this configuration, the monitoring instruments — stationed on the motor casing and attached to the motor wiring cabinet wiring — were tasked with recording vibration signals and current signals during the motor’s operational period, thereby accruously capturing essential data indicative of the system’s functional state.

In this study, we used a dataset consisting of vibration and current signals collected from induction motors operating at two different rotational speeds: 1800 r/min and 2400 r/min. The signals were collected using high-precision accelerometers (model: PCB 356A16) and current clamps (model: Fluke i200s) with low noise levels (accelerometer noise floor: 0.0003 g RMS, current clamp accuracy: ± 1%) to ensure accurate data capture. The dataset includes five different states: standard functioning (F1), bent rotor anomaly (F2), broken rotor complication (F3), rotor imbalance issue (F4), and bearing impairment (F5). Each state has 1000 batches of data, making a total of 5000 batches for each rotational speed. The faults were simulated at different severity levels to capture a range of fault conditions. For instance, the bent rotor anomaly (F2) was simulated with varying degrees of bending (mild: 0.1 mm, moderate: 0.5 mm, severe: 1.0 mm), and the broken rotor complication (F3) was simulated with different numbers of broken bars (1, 2, or 3 bars). Additionally, the data were collected under various load conditions (0%, 25%, 50%, 75%, and 100% of the motor’s rated load) to ensure the model’s robustness to different operational environments. To maintain class balance, each fault class has an equal number of samples (1000 batches per state), and the training and testing sets were split based on different time periods (training: first 80% of data collection period, testing: last 20%) to prevent data leakage.

**Fig 11:**
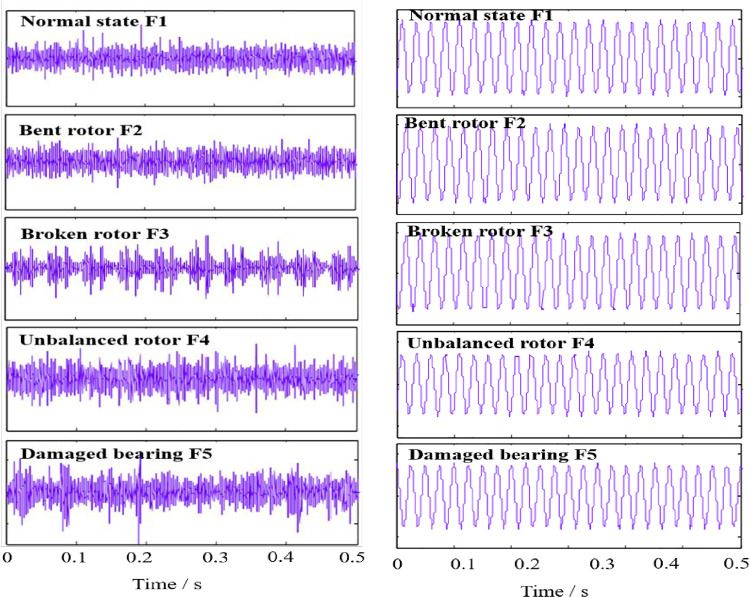
Time-domain waveform of vibration signal and current signal. (a) Vibration signal (b) Current signal.

Fig 11 delineates the temporal domain waveforms illustrating the vibration and current signals under diverse healthy conditions, thereby offering a visual representation that encapsulates the specific signal patterns inherent to each state of the induction motor, aiming to furnish a more nuanced understanding and facilitate a meticulous analysis of the collected data.

### 4.2 Dataset pre-processing

First, the collected induction motor stator current and vibration signals are constructed as a data set, of which 80% are randomly selected as the training set and 20% data as the test set. Then, the data are denoised using a denoising autoencoder network. Finally, the noise-reduced data are subjected to signal-to-image conversion.

Since the number of convolution kernels of the pooling layer used in the 2D CNN model is 2 × 2, the size of each layer of image features will be reduced by half after pooling, so the best value for the edge length *N* of 2D grayscale images is 2^*n*^, such as 16, 32, 64, 128. Considering that the size and number of input images will have certain influence on the fault recognition accuracy and efficiency, therefore, in this paper, after the CNN model training, the selected image size is 64 × 64.

### 4.3 Experimental results and comparative analysis

To verify the effectiveness of the proposed multi-source information fusion model for induction motor fault diagnosis, the fault diagnosis models constructed by the multi-source information fused by the proposed method, the multi-source information fused by the traditional method, the vibration signal and the stator current input are presented respectively. Among them, the traditional multi-source information fusion method is to serially superimpose multiple signals into one signal. The vibration features are the time domain features of the vibration signal. The current features are the wavelet packet entropy features of the current signal. To eliminate the effect of chance errors, 10 trials were conducted for each method. Tables 3 and 4 give the average values of the results of the 10 trials for the different methods on the 1800 and 2400 r/min test sets.

**Table 3:** Troubleshooting results at 1800r/min.

Feature Type	Fault identification rate/%					
F1	F2	F3	F4	F5	Avg.
Vibration Feature	82.1	69.3	58.7	70.4	95.6	75.2
Current Feature	55.6	68.2	69.3	80.6	83.3	71.4
Traditional multi-source information fusion	89.7	90.2	85.3	90.7	91.2	89.4
Proposed multi-source information fusion	98.3	100.0	96.9	100.0	100.0	99.0

**Table 4:** Troubleshooting results at 2400r/min.

Feature Type	Fault identification rate/%					
F1	F2	F3	F4	F5	Avg.
Vibration Feature	70.6	66.2	59.5	98.3	93.9	77.7
Current Feature	75.3	77.4	70.8	85.7	86.8	79.2
Traditional multi-source information fusion	77.3	79.6	75.2	97.6	95.7	85.1
Proposed multi-source information fusion	85.5	98.2	90.1	100.0	100.0	94.8

As can be seen from Tables 3 and 4, the average fault identification rates of the heterogeneous fusion features at the 2 speeds reach 99.0% and 94.8%, respectively, which are significantly higher than the fault identification rates of the original features of the single signal and the corresponding kernel principal features. This indicates that compared with the single signal feature, the kernel principal element fusion feature of heterogeneous information makes full use of the redundant and complementary information of the heterogeneous sources and the nonlinear relationship between the original feature data to obtain more comprehensive information characterizing the operating status of the equipment and improve the recognition capability of the classifier. In addition, the proposed multi-source information fusion method is also significantly better than the traditional fusion model in terms of diagnostic performance. Multi-sensor signal fusion can provide richer fault diagnosis information, and the proposed fusion method has lower standard deviation and more stable diagnosis results.

**Fig 12:**
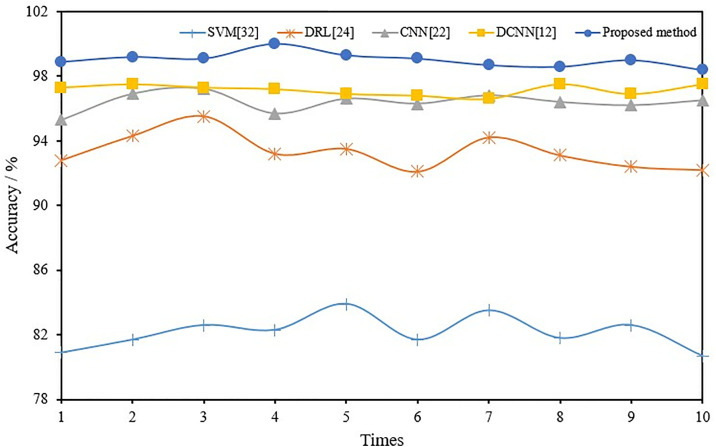
Fault-diagnosis-accuracy curves for different methods (1800r/min).

**Fig 13:**
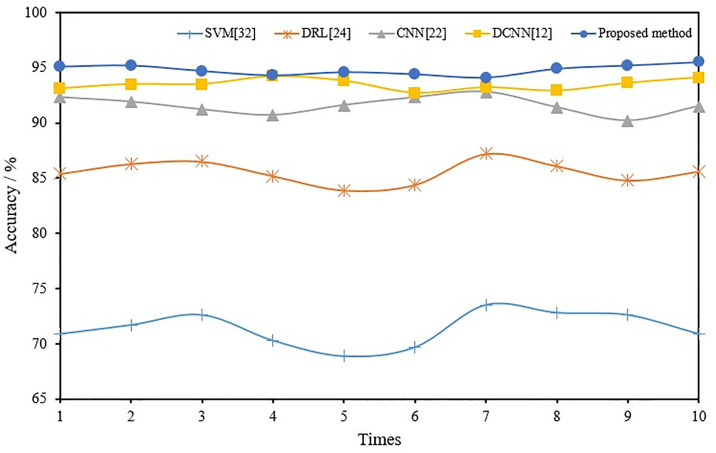
Fault-diagnosis-accuracy curves for different methods (2400r/min).

To further demonstrate the superiority of adaptive CNN, the fault recognition capabilities of CNN [22], DCNN [12], DRL [24], and SVM [32] were compared in fusion mode. To eliminate the effect of chance errors, 10 trials were conducted for each method. Tables 5 and 6 provide the average values of 10 test results using different methods at rotor speeds of 1800 and 2400 r/min. Figs 12 and 13 show the comprehensive recognition rate of the F1-F5 state of the induction motor during each test on the 1800 and 2400r/min test sets.

**Table 5:** Fault diagnosis results of 5 methods (1800r/min).

Feature Type	Fault identification rate/%					
F1	F2	F3	F4	F5	Avg.
SVM [32]	80.3	82.2	79.5	85.5	83.6	82.2
DRL [24]	92.6	95.1	88.3	96.0	94.2	93.3
CNN [22]	95.8	98.7	91.2	98.5	97.7	96.4
DCNN [12]	96.5	99.2	93.3	98.9	98.1	97.2
Proposed method	98.3	100.0	96.9	100.0	100.0	99.0

**Table 6:** Fault diagnosis results of 5 methods (2400r/min).

Feature Type	Fault identification rate/%					
	F1	F2	F3	F4	F5	Avg.
SVM [32]	69.2	73.7	67.6	75.2	71.3	71.4
DRL [24]	78.8	86.6	80.2	90.6	91.1	85.5
CNN [22]	81.8	96.7	86.9	96.5	95.9	91.6
DCNN [12]	83.4	97.1	89.5	98.4	99.0	93.5
Proposed method	85.5	98.2	90.1	100.0	100.0	94.8

As found in Tables 5 and 6 and Figs 12,13, the proposed method achieves an average accuracy of 99.0% and 94.8% on the 1800 and 2400 r/min test sets compared to other machine learning methods. Its diagnostic performance is generally better than traditional CNN, DCNN and other shallow learning networks, indicating that the proposed adaptive CNN-based fault diagnosis method can effectively extract representative fault features from multiple sources of information and improve the fault classification accuracy.

In addition to comparisons with traditional machine learning and deep learning models, we further compare our proposed method with several existing multi-source information fusion-based fault diagnosis methods to highlight its advantages in both methodology and performance. Specifically, we conducted a comparative analysis with two multi-source information fusion-based fault diagnosis approaches: (1) a fusion correlation spectrum approach combining vibration and current signals [24]; and (2) a 1D-CNN-based method using combined vibration and instantaneous speed signals [33]. These models represent key developments in multi-sensor fusion strategies for induction motor fault diagnosis. Table 7 summarizes the comparative results on the same dataset (at 1800 r/min), using the average fault recognition accuracy across all categories.

**Table 7:** Comparison with other multi-source fusion-based fault diagnosis methods.

Method	Avg. Accuracy (%)
Fusion correlation spectrum (current + vibration) [24]	91.2
1D-CNN with vibration and speed fusion [33]	94.3
Proposed	99.0

As shown, our proposed method achieves the highest diagnostic accuracy of 99.0%, outperforming other fusion strategies by a significant margin. This result confirms that our integration of correlation-based data layer fusion and adaptive CNN-based feature fusion offers superior representational capacity and classification robustness.

## 5 Conclusion

In the present study, we introduce an innovative induction motor fault diagnostic technique that leverages multi-source information fusion and adaptive CNN to address the limitations of single-sensor approaches. Our approach effectively utilizes vibration and current signals, preprocessed through a denoising autoencoder, and integrates homogeneous and heterogeneous data at the data and feature levels, respectively. As a pivotal component of this methodology, we advocate for the implementation of an adaptive CNN, which has been tailored to facilitate the precise classification of a spectrum of induction motor faults with enhanced accuracy. Empirical results significantly outperform traditional methods and other advanced multi-source fusion techniques, underscoring the method’s potential as a robust diagnostic tool.

Despite these achievements, the proposed method has several limitations that warrant consideration. First, its generalizability to different motor types, operational environments, or unseen fault scenarios remains to be fully explored, as the current validation is based on specific datasets and fault types. In addition, the “black-box” nature of deep learning models, including CNNs, poses challenges for interpretability, which is crucial for gaining trust and facilitating adoption in industrial applications. To address these limitations and further enhance the method’s applicability, future work will focus on several key areas. We intend to expand our dataset by incorporating extra fault types, including eccentricity faults, stator inter-turn short circuits, compound faults, and variable load conditions, to assess the scalability and adaptability of the proposed diagnostic framework. Additionally, efforts will be made to develop interpretability mechanisms, such as attention maps or feature importance scores, to provide insights into the model’s decision-making process.
